# Therapeutic utility of glucocorticoids and antihistamines cotreatment. Rationale and perspectives

**DOI:** 10.1002/prp2.530

**Published:** 2019-12-20

**Authors:** Carlos D. Zappia, Federico Monczor

**Affiliations:** ^1^ Facultad de Farmacia y Bioquímica Universidad de Buenos Aires Buenos Aires Argentina; ^2^ Instituto de Investigaciones Farmacológicas (ININFA) CONICET ‐ Universidad de Buenos Aires Buenos Aires Argentina

**Keywords:** antihistamines, drug association, glucocorticoid receptor, glucocorticoids, histamine H_1_ receptor, inflammation

## Abstract

Antihistamines and glucocorticoids (GCs) are often used together in the clinic, in several inflammatory‐related situations. Even though there is no clear rationale for this drug association, the clinical practice is based on the assumption that due to their concomitant antiinflammatory effects, there should be an intrinsic benefit in their coadministration. Our group has studied the molecular interaction between the histamine H_1_ receptor and the glucocorticoid receptor (GR) signaling pathways, showing an enhancing effect on GC‐induced GR transcriptional activity induced by antihistamines. We hypothesize that the existence of this synergistic effect could contribute in reducing the GCs clinical doses, ineffective by itself but effective in combination with an antihistamine. This could result in a therapeutic advantage as the GC‐desired effects may be reinforced by the addition of an antihistamine and, as a consequence of the dose reduction, GC‐related adverse effects could be reduced or at least mitigated. Here we discuss the potential therapeutic applications of this cotreatment seeking to evaluate its usefulness, especially in inflammatory‐related conditions.

**Abbreviations**
AD: atopic dermatitis
AP‐1: activator protein 1
AR: allergic rhinitis
AZE: azelastine
DEX: dexamethasone
FLU: fluticasone
GC: glucocorticoid
GR: glucocorticoid receptor
H_1_R: histamine H1 receptor
HA: histamine
NF‐κB: nuclear factor kappa B
PPAR: peroxisome proliferator‐activated receptor
TA: transactivation
TR: transrepression
β_2_R: beta‐2 adrenergic receptor

## 1 GLUCOCORTICOIDS

Glucocorticoids (GCs) belong to the family of steroid hormones and are synthesized in the fascicular zone of the adrenal cortex, whose primary function is to maintain body homeostasis. The antiinflammatory properties of GCs came to prominence when cortisol was used in the suppression of the clinical manifestations of rheumatoid arthritis 60 years ago, and they have been used in medicine since then.^1^ For this contribution, in 1950 the Nobel Prize in Physiology or Medicine was awarded to Kendall and Reichstein. The clinical demand for GCs rapidly increased and numerous compounds were synthesized. Among them, prednisone, prednisolone, dexamethasone, and triamcinolone first appeared in the 1950s and are still in use.^2^

GCs exert their antiinflammatory effects by binding to the glucocorticoid receptor (GR) and by modulating gene expression. In the absence of its ligand, the GR is predominantly located in the cytoplasm, in a complex containing multiple proteins such as the chaperones Hsp90, Hsp50 and Hsp70, and the immunophilins FKBP51 and FKBP52. Upon ligand binding, the GR undergoes a conformational change resulting in dissociation from this multiproteic complex and nuclear translocation.^3^ Once in the nucleus, GR can positively regulate (or transactivate) anti‐inflammatory genes expression, mainly by binding directly to promoter regions of target genes, or negatively regulating, or transrepressing, the expression of proinflammatory genes, mostly by physical interaction with other transcription factors such as nuclear factor κB (NF‐κB) or activator protein 1 (AP‐1). A growing body of evidence has shown some rapid effects on inflammation, which would not be mediated by changes in gene expression. However, much remains to be clarified in relation with the role of these mechanisms in their antiinflammatory action.^4^

Nowadays, GCs are among the most widely prescribed drugs in clinical practice because of their strong antiinflammatory and immunosuppressive effects. They represent a standard therapy for several autoimmune, inflammatory, and allergic disorders, such as rheumatoid arthritis, asthma, lupus erythematosus, inflammatory bowel disease and transplant rejection.^5^ Nevertheless, therapy is commonly associated with a large amount of serious adverse effects including osteoporosis, dyslipidemia, body fat redistribution, muscle wasting and atrophy, insulin resistance, glucose intolerance and even diabetes.^6^ The occurrence and severity of the GC are determined by the duration, dosage and dosing regime, the nature and its route of administration as well as by the individual susceptibility of each patient.^7^ The main challenge always was, and still remains, to improve their antiinflammatory actions while minimizing their adverse metabolic effects.^8^

## 2 TRANSACTIVATION VS TRANSREPRESSION

As mentioned, to maintain homeostasis, GCs act on almost every cell of the human body, regulating physiological processes including intermediary metabolism, immune function, skeletal growth, cardiovascular function, reproduction, and cognition. According to this, it becomes nearly impossible to separate the antiinflammatory effects from the metabolic undesired effects. The hypothesis whereby the antiinflammatory effects of GCs are mainly determined by the transpression (TR) of GR of pro‐inflammatory transcription factors, while their adverse effects are induced by transactivation (TA) of genes, has led to the identification of dissociated GCs or selective GR agonists (SEGRAs). RU24782, RU24858, and RU40066 have been shown to reduce TA activity but still retain strong antiinflammatory activity. Unfortunately, to date, only two compounds have been evaluated in clinical trials, Mapracorat for topical applications and Fosdagrocorat for rheumatoid arthritis treatment.^9^, ^10^ Their lack of efficacy is probably due to the importance of TA in resolving inflammation. The continuous identification of new TA‐dependent genes with antiinflammatory properties supports this notion and reflects the importance of TA in these processes. In addition, the development of those initially promising compounds was slowed down due to the occurrence of clinical bone‐related adverse effects. Several studies have proved that GCs’ adverse effects such as osteoporosis or muscular atrophy, also occur through TR.0.^11^ In this context, since TA and TR involve both antiinflammatory and GR‐related side effects, dissociating them by dissociating TA and TR has become a chimera.

## 3 GR MODULATORS

Because of the complexity of the GR biology it has been hypothesized that different GR conformations would lead to different transcriptional profiles and ultimately to different pharmacological outcomes.^12^, ^13^ This has driven the search towards ligands that activate the GR in specific conformations to selectively modulate its function (SEGRMs), broadening from steroidal to nonsteroidal scaffolds. One example is the nonsteroidal Compound A, which was classified as a selective GR modulator because it was able to partially interfere selectively with GC‐activated TA‐related gene expression eliciting a different conformation of GR in relation to classic GCs.^14^ Derived from this, the two most extreme modulators would be those that induce only GR monomer (SEMOGRAMs) or dimer (SEDIGRAMs) conformations. Since the treatment of different diseases will mainly benefit from monomeric or dimeric GR actions, it has been hypothesized that SEMOGRAMs may be useful in chronic inflammatory diseases, while SEDIGRAMs may be useful in acute inflammatory conditions. However, the assumption that different GCs may lead to different GR dimerization degrees and to different transcriptional activities requires further validation and consequently, there is a need for more clinical evidence to evaluate the GR dimerization hypothesis.^15^

## 4 COMPLEMENTARY THERAPIES

Another approach to deal with GC‐related adverse effects is the addition of a different drug to a corticoid‐based therapy, aiming to reduce the dose of corticoid and consequently its side effects. As mentioned before, the occurrence and severity of the secondary effects are mainly determined by the duration and dosage of the treatment.^7^ Asthma is a well‐known example where different add‐on therapies are recommended. GCs are commonly used together with β2‐adrenoceptor (β_2_R) agonists, theophylline or anti‐leukotrienes, being the first one, the most effective combination.^16^ Due to its bronchodilator effect, it was suggested that the addition of β_2_R agonists may have complementary actions to GCs on the physiopathology of asthma. At the molecular level, it has been described synergic, or at least additives effects, since it was reported that β_2_R agonists increase GC‐induced GR nuclear localization and its transcriptional activity.^17^ More recent and promising, but still preclinical, strategies to circumvent GCs’ side effects involve the stimulation of the crosstalk between the GR and the peroxisome proliferator‐activated receptor (PPARs), as their signaling pathways have overlapping and complementary roles in many tissues. By combining GR with PPARα stimulation, their antiinflammatory effects might be additive, but not their side effects.^15^

## 5 ANTIHISTAMINES

The first antihistamines have arisen through the finding of the antihistaminic effects associated with piperoxane by Daniel Bovet and Anne‐Marie Staub in 1937. For his work on antihistamines and curare, Bovet was awarded the Nobel Prize in Physiology or Medicine in 1957. However, it was more than 20 years earlier when Sir Henry Dale demonstrated the effects of histamine (HA) on the stomach's smooth muscle and the respiratory tract, as well as its vasopressor and shock‐related effects when injected into animals.^18^ The years following saw the emergence of several reports leading to the establishment of the role of HA in allergic and anaphylactic processes. These findings triggered the search for antagonists that were able to prevent the pathological effects of HA at the Pasteur Institute, where Bovet worked. Based on the common characteristics between HA, acetylcholine and adrenaline, in 1937 related compounds started to be explored leading to the discovery of piperoxane and its associated compounds, capable of preventing the lethal effects induced by HA administration in guinea pigs.^19^

In 1942 the first antihistamine was approved for its use in humans, and nowadays antihistamines represent the largest group of medicines used in allergic disorders, with more than 45 clinical antihistamines available worldwide. Initially classified as antagonists of the histamine H_1_ receptor (H_1_R), they have been reclassified as inverse agonists, capable of stabilizing the inactive form of the H_1_R.^20^, ^21^, ^22^ Through this mechanism of action and also by antagonizing the effects of HA at the H_1_R, these drugs interfere with the allergic‐inflammatory processes, becoming the second‐generation antihistamines, medications of choice in patients with allergic rhinitis (AR), conjunctivitis allergic and urticaria. Antihistamines are also used in disorders where there is no strong clinical evidence regarding their efficacy, such as atopic dermatitis (AD), anaphylaxis, nonallergic angioedema, otitis media, sinusitis, insomnia, anxiety, migraine and other vestibular disorders.^23^ Even when they are indicated for patients with allergic rhinitis with concomitant asthma, they are not used in patients with asthma.^24^

## 6 ANTIHISTAMINES AND GLUCOCORTICOIDS

Antihistamines, as well as glucocorticoids, are widely used for the treatment of allergic and inflammatory conditions. It is important to highlight that the targets with the greatest number of approved drugs are their receptors, the GR and the H_1_R.^25^ Even when there is no clear rationale for this drug association, they are commonly used together in a number of inflammatory‐related clinical situations. This clinical practice has been stablished based on the assumption that there should be an intrinsic benefit in their coadministration due to their antiinflammatory effects.^26^

It has been shown that the combination of antihistamines and corticoids is the most widely used choice to treat all types of AR.^27^ Several clinical trials have shown the benefits of the combination of the antihistamine azelastine (AZE) and the corticoid fluticasone (FLU), resulting in the patent granting of the first steroid plus antihistamine nasal spray (Dymista®) along with two other steroid combinations.^28^ Recently published guidelines also position AZE + FLU as a first‐line treatment for moderate to severe AR, in preference to an inhaled GC.^29^ The 2016 update to the Allergic Rhinitis and its Impact on Asthma (ARIA) guidelines recommends this combination for seasonal AR.^30^ To date, AZE + FLU is the only combination available for the treatment of AR. Its efficacy is ascribed to the antiallergic and antiinflammatory effects of the antihistamine in the early phase reactions and the potent inhibition of the late‐phase allergic reactions by the corticoid, resulting in significant additive effects.^31^ Nevertheless, nothing is known about the molecular interaction between the intracellular signaling pathways of both ligands. Pathophysiological gaps related to this combined therapy claim for further study of the molecular mechanisms of action.^32^

Likewise, AD therapy is another example of the concomitant use of antihistamines and corticoids. This inflammatory and chronic skin disease is widely treated with topic corticoids, even though the treatment presents many adverse effects and its efficacy and mechanism of action are not well understood. Antihistamines have been used jointly with corticoids for AD treatment albeit little is known about its usefulness or potential interactions.^33^ On the preclinical level, the synergistic effects of antihistamines and corticoids in an animal model of AD have been evaluated, finding that the antihistamine olopatadine enhanced the corticoid prednisolone antiinflammatory effect, leading to the conclusion that this drug combination could be useful to treat AD, although the mechanism underlying the synergism is also unknown.^34^

## 7 CELL SIGNALING PATHWAYS CROSSTALK

The ability of a cell to answer internal and external stimuli is a vital property. Continuously, each cell of a multicellular organism receives a vast number of messages, which are integrated through a limited number of signaling pathways to produce complex cellular responses. These pathways not only transmit the messages but also process and codify them. This reflects the astonishing properties of the biological systems to detect stimuli and adapt their biochemical machinery upon a wide range of situations to maintain homeostasis. This feature compels to conceive cell communication as a complex interaction network as of their single components.^35^

The classical depiction of linear and discrete signaling pathways has been replaced by a big amount of multiple interconnected networks, leading to a holistic conception of cell communication. However, not all interactions have biological or clinical relevance. In this sense, it is desirable, from a pharmacological standpoint, to investigate the crosstalk between signaling pathways that could lead to new ways of understanding pathologies and therapies.^36^

## 8 GPCRS‐GR CROSSTALK

Modulation of the GR's activity has been extensively investigated since the first report of the receptor appeared in 1967,^2^ showing that its activity can be modulated by different signaling pathways. In contrast, there are few reports, documenting the crosstalk between GR and G‐protein‐coupled receptors (GPCRs) signaling at the molecular level. Acting on the β2‐adrenergic receptor, epinephrine and nor‐epinephrine enhances GR's activity through Gβγ subunits, PI3K and PKB pathway, but independently of its classical effector PKA.^37^ Somatostatin suppresses GR's activity directly in the cell nucleus through the binding and nuclear translocation of the Gβγ subunits along with the GR.^38^ Finally, it has been reported that melatonin suppresses GR's transcriptional activity through MT1 receptor‐coupled Gαi subunit,^39^ inhibits the synthesis of the GR's messenger RNA,^40^ prevents GC‐induced apoptosis,^41^ prevents GR's nuclear translocation by blocking the dissociation from its chaperone Hsp90,^42^ and affects its interaction with the coactivator TIF‐2.^43^ It has been also described that the orphan GPCRs, GPR30 and GPR50, modulate GR's activity, the first one by inhibiting the expression of the coactivator TIF‐2 and the second one, by interacting with coactivator TIP‐60.^44^, ^45^

## 9 H_1_R‐GR CROSSTALK

**Figure 1:**
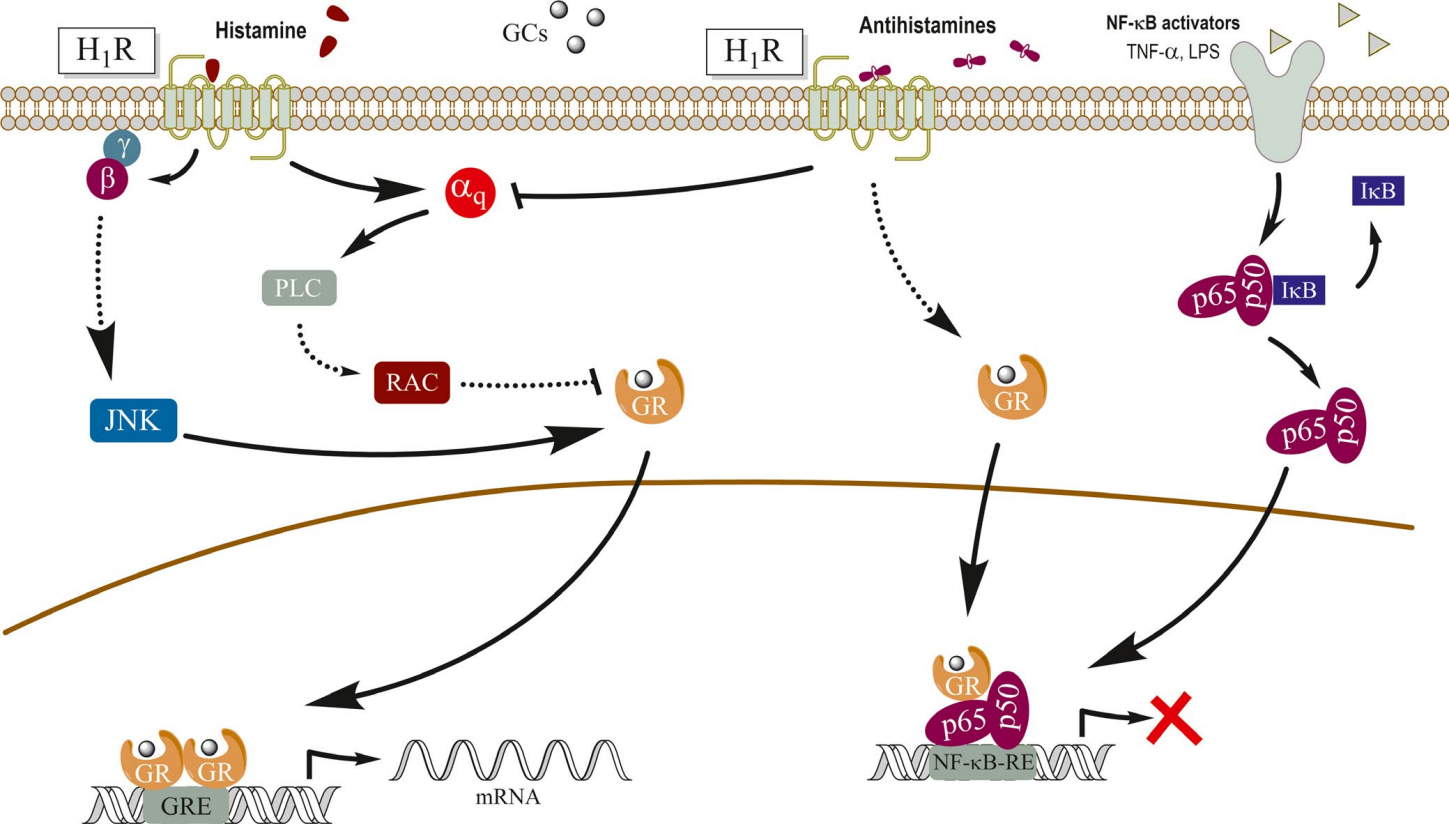
Mechanism of action proposed for the crosstalk between H_1_R and GR signaling pathways. Activation of the H_1_R triggers a complex dual regulatory mechanism on GR activity, involving both Gαq and Gβγ G‐protein subunits. While the Gαq subunit has an inhibitory effect via a PLC‐RAC‐mediated pathway, Gβγ enhances GR activity via JNK. While activation of the H_1_R by histamine resulted in a composite potentiating effect, inactivation of the Gαq‐PLC pathway by H_1_R inverse agonists resulted in a potentiation of GR activity. The enhancing effect of antihistamines occurs both for GR‐mediated transactivation of GRE‐dependent genes and for GR‐mediated transrepression of genes regulated by NF‐κB (p65‐p50). Solid lines indicate direct effects; dashed lines indicate indirect effects. Lines ending with arrowheads or bars indicate activating or inhibitory effects, respectively. GR: glucocorticoid receptor; H_1_R: histamine H1 receptor; NF‐κB: nuclear factor kappa B; TNF‐α: tumor necrosis factor‐alpha; LPS: lipopolysaccharide; PLC, phospholipase C; JNK: Jun kinase; I‐κB: inhibitor kappa B; GRE: glucocorticoid response element; NF‐κB‐RE: NF‐κB response element

Given the pharmacological importance and clinical relevance of antihistamines and corticoids combination therapies, our group focused on how H_1_R signaling pathways modulate GR‐mediated transcriptional activity. Our results show a complex dual regulation of GR activity, consisting of an enhancing effect involving the G‐protein βγ subunits and Jun kinase (JNK) and a parallel inhibitory effect mediated by the canonical Gαq‐PLC‐Rac pathway. An overall activating effect is observed when the H_1_R is activated by its natural agonist, histamine, which is the result of a simultaneous triggering of both pathways. Conversely, when H_1_R is bound by inverse agonists, the inhibitory Gαq‐PLC‐Rac pathway is repressed, also resulting in an enhancement of GR‐mediated transcriptional activity. This paradoxical observation that the natural full agonist as well as the H_1_R inverse agonists potentiate GR activity can be explained in terms of the mechanism of action proposed (Figure 1). The existence of antihistamines’ enhancing effect was observed both for GR transactivation and transrepression processes in heterologous expression systems through reporter gene assays, and it was replicated in physiopathological cell models by measuring the expression of endogenous inflammatory‐related genes.^26^

## 10 PHARMACOLOGICAL HYPOTHESIS

Based on the previously described findings we hypothesize that the enhancement of the GR transcriptional activity by antihistamines could allow for a reduction in the GCs doses normally used in the clinic. This reduced dose would be ineffective by itself but effective in combination with an antihistamine, possibly resulting in new therapeutic strategies to treat different conditions. Since the duration and dosage of treatment with GCs determine the occurrence and severity of its adverse effects, a reduction of its dose may result in an improvement of its undesired effects without compromising its therapeutic efficacy.

## 11 POTENTIAL SCENARIOS

The modulation described should only be possible in those cell types that coexpresse the H_1_R and the GR, allowing the interaction of their signaling pathways. Among them, endothelial cells, dendritic cells, monocytes, neutrophils, B and T lymphocytes, and glial cells are examples of GR and H_1_R coexpression.^46^, ^47^ The existence of these cell types coexpressing both receptors suggests that this drug association strategy may have many implications in several systems or organs such as the immunological system, lungs, skin or the central nervous system. Particularly, the inflammatory‐allergic pathologies are especially relevant in most of the cell types belonging to the immunological system. The role of dendritic cells in asthma and allergic rhinitis has been studied for the last fifteen years.^48^ For its part, monocytes, lymphocytes and neutrophils have an important role in chronic inflammation of the airways in pathologies such as asthma and chronic obstructive pulmonary disease.^49^, ^50^, ^51^ Likewise, endothelial cells have a crucial role in the development and worsening of allergic disorders.^52^

## 12 ASTHMA

Besides providing rational support to many of the current therapies where corticoids and antihistamines are used in combination, the description of the GR and H_1_R signaling pathways crosstalk can lead to the development of new therapeutic strategies where cotreatment can be justified. Asthma is a high morbidity and mortality chronic inflammatory disease, and corticoids are currently the most effective therapy, but there is a lack of efficacy of antihistamines in controlling the symptoms.^16^, ^53^ Therapy with GCs is commonly associated with severe adverse effects, especially at high doses in long‐term treatments, which often limits their use.^7^ This, together with the existence of asthma patients unable to control their symptoms, generates the need for new therapeutic strategies.^54^ We have studied the effects of the combined administration of dexamethasone (DEX) and the antihistamine azelastine (AZE) in an allergen‐induced murine model of asthma. Our results indicated that the combination of AZE and DEX in a dose ineffective by itself can improve allergic lung inflammation as shown by a decrease in eosinophils in bronchoalveolar lavage, reduction of peribronchial and perivascular infiltrates and mucin‐producing cells, diminished serum levels of allergen‐specific IgE and IgG1, and a reduction in the expression of inflammation‐related genes IL‐4, IL‐5, Muc5AC and Arginase I in the lung.^55^ The finding that AZE potentiated DEX‐induced effects in vivo, leads us to suggest that this potentiation might allow for a reduction of the GC therapeutic dose needed, supporting the consideration of antihistamines as add‐on drugs in GC‐mediated antiasthmatic therapies. The potential benefits of the cotreatment consist in a reduction of GC‐related adverse effects without losing therapeutic efficacy.

## 13 NEUROINFLAMMATION

Neuroinflammation comprises every inflammatory process that occurs in the CNS and involves distinct cell types and mediators depending on its onset and progress. Astrocytes and microglia are the main cells resident in the CNS responsible for the inflammatory and immunological responses. Their activation induces inflammation and release of several mediators including cytokines, chemokines or growth factors that originate and sustain the inflammatory response.^56^In general, acute and transient inflammation is a beneficial process that induces an adaptative response to protect the CNS from an aggression or an injury, while chronic and long‐term inflammation can lead to the production of neurotoxic mediators. The rise in pro‐inflammatory cytokine expression from astrocytes and microglia inside the brain results in neuroinflammation that ends in neurodegeneration.^57^, ^58^ The relationship between neuroinflammation and neurodegeneration has been intensely reviewed. In the last few years, evidence points to neuroinflammation as an effector in neuronal dysfunction, cell death and tissue damage.^59^, ^60^ Moreover, it has documented the association between neuroinflammation and neurodegenerative diseases such as Alzheimer's or Parkinson's diseases among others.^61^, ^62^ The brain has been distinguished as the main target of neuroprotective strategies and therefore, the identification of glial inflammatory regulators has been recognized for their therapeutic value related to Alzheimer's disease and other neuropathologies.^63^ However, although a lot of evidence supports the relationship between neuroinflammation and neurodegeneration, so far there is not an effective therapeutic strategy based on this approach. Non‐steroidal anti‐inflammatory drugs, omega‐3 polyunsaturated fatty acids, and immunological antagonists have been clinically evaluated, concluding that even though the therapeutic strategy is valid, the clinical efficacy is limited.^64^ Identification of new pharmacological targets addressing neuroinflammation will deepen the knowledge of these processes and its relation to neurodegenerative diseases, and may lead to development of new therapies to treat neuroinflammatory‐related conditions, many of which lack an adequate or efficient therapy. In this sense, there is a potential therapeutic utility for corticoids and antihistamines cotreatment in neuroinflammatory contexts. GCs are well‐known to be released as a feedback mechanism to quench an inflammatory response, however, more recently they have been shown to have proinflammatory effects. Furthermore, the effects of chronic exposure to GCs in the brain have been suggested to be more complex than its acute antiinflammatory effects in the periphery. The roles of histamine and its receptor ligands are also complex as well.^65^ We hypothesize that antihistamines can modulate GCs effects in the CNS towards an antiinflammatory action, establishing the potentiality of their receptors as targets for the treatment of neurodegenerative diseases.

## 14 GC‐RELATED ADVERSE EFFECTS

Both GCs’ therapeutic and adverse effects are on‐target and exerted through gene modulation by the GR. Many of the adverse effects mainly involve TA processes (glaucoma, hypertension, diabetes) while others are the result of TR of genes induced by the GR (hypothalamic‐pituitary‐adrenal axis suppression, infections susceptibility). Some of them involve both mechanisms (osteoporosis) and many others are still not completely elucidated (gastrointestinal bleeding and peptic ulcer).^6^ Since antihistamines could enhance GC‐induced GR activity both for TA and TR, it is critical to address the potential modulation of GR adverse effects by antihistamines. No study is complete if the potential adverse effects are ignored. In consequence, the adverse effects described before and their underlying molecular mechanisms must be considered when evaluating the safety of antihistamine and corticoid cotreatment.

## 15 CONCLUSIONS

The notion supported by molecular evidence obtained in vitro and in vivo, that the GR transcriptional activity can be modulated by H_1_R signaling pathways and that this modulation may have therapeutic relevance, provides the basis for the pharmacological hypothesis proposed herein and invites to go further into the study, aiming to address the potential therapeutic application of corticoids and antihistamines cotreatment in different settings. Given that both ligands are often used together in several clinical scenarios, it is important to investigate the molecular consequences of this drug association, especially when multiple pathways are modulated. To understand the shared mechanism of action of such widely used drugs is crucial to improve their specificity and safety, giving rationale to this commonly associated drug combination.

## DISCLOSURE

The authors declare that the research was conducted in the absence of any commercial or financial relationships that could be construed as a potential conflict of interest.

## Data Availability

The data that support the findings of this study are available from the corresponding author upon reasonable request.
